# Dynamic Response Mechanism of Bedding Slopes with Alternatively Distributed Soft and Hard Rock Layers Under Different Seismic Excitation Directions: Insights from Numerical Simulations

**DOI:** 10.3390/ma17235939

**Published:** 2024-12-04

**Authors:** Yuanyuan Zhou, Fei Zhao, Zhenming Shi

**Affiliations:** 1 College of Transportation Engineering, Nanjing Tech University, Nanjing 211816, China; 2 Department of Geotechnical Engineering, College of Civil Engineering, Tongji University, Shanghai 200092, China

**Keywords:** bedding slope, soft and hard rock layers, three-directional seismic waves, acceleration amplification factor, response mechanism

## Abstract

The issue of slope stability in earthquakes has become increasingly prominent with the construction of many infrastructure projects such as highways, bridges, and tunnels. To explore the dynamic response characteristics of bedding rock slopes in an earthquake, the three-dimensional dynamic finite-difference method (TDD-FDM) in this study is used to establish simplified rock slope models, taking a bedding rock slope with alternatively distributed soft and hard rock layers in Yunnan, China as a prototype. The dynamic response mechanism of layered rock slopes containing different thicknesses, locations, and quantities of soft rock layers was studied under different excitation directions of seismic waves. The main findings are that the propagation of seismic waves at different rock layer structures has directionality, which causes the strongest seismic response to be all located in the upper or middle parts of the slope; the influence of rock structures on seismic response in layered rock slopes is in the order of thickness > quantity > location; the acceleration amplification effect of a slope under multi-directional seismic wave excitation exhibits the phenomena of differential amplification and coupling amplification; and the acceleration amplification factors of a slope with increasing peak ground acceleration from 0.05 g to 0.20 g show two trends: increasing–decreasing and continuous increasing. The findings of this study can be a reference for studying the dynamic response of rock slopes in strong earthquakes.

## 1. Introduction

**Figure 1:**
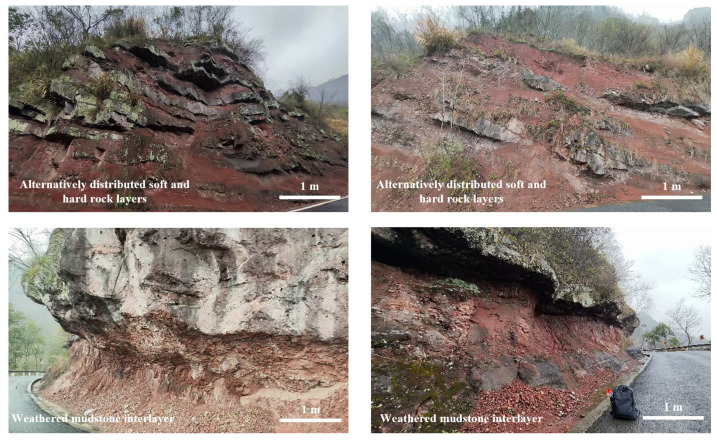
Rock slopes with soft rock layers and weak interlayers (Ludian, Yunnan, China).

With the implementation of the important strategy of “one belt and one road” in China, the issue of slope geological hazards is a critical factor impacting the safety of geotechnical engineering infrastructures like highways, bridges, and tunnels, particularly in mountainous regions, which are prone to severe earthquakes. Earthquakes trigger numerous slope geological hazards, resulting in substantial economic losses and human casualties [1,2,3,4,5,6]. According to the investigations of slope disasters after earthquakes, the failure of layered rock slopes caused by earthquakes is the most common and serious, including typical landslide events such as Tangjiashan landslide, Wangjiayan landslide, and Hongshiyan landslide [7,8,9,10,11]. This is due to a significant difference in the response characteristics of layered rock slopes under seismic loads compared to static loads. On one hand, natural rock slopes contain a large number of structural planes with different characteristics, such as bedding, joints, fissures, weak interlayers, veins, and faults (Figure 1), which determines the heterogeneity and anisotropy of layered rock masses, leading to increasingly complex engineering geological conditions of slopes and unclear dynamic response patterns of rock slopes [12,13,14,15,16,17,18,19]. On the other hand, seismic loads are complex and random, which makes it more difficult to evaluate the dynamic stability of layered rock slopes. Therefore, the dynamic response and stability of rock slopes under seismic loads have increasingly become a key research issue in the fields of geotechnical engineering and engineering geology. In view of this, scholars have conducted extensive research on the dynamic response and stability of layered rock slopes through various technical methods such as field investigations, model experiments, numerical simulations, and theoretical analysis, especially on those containing soft rock layers and weak interlayers [20,21,22,23,24,25,26,27,28].

Researchers have studied the effects of soft rock and weak interlayers on the dynamic response of slopes, including factors such as strength, location, quantity, thickness, and inclination angle. In terms of the location of weak interlayers or soft rocks, Feng et al. [9] found that the closer the soft rock is to the upper part, the severer the slope vibration damage. Liu et al. [29] studied that the dynamic response of the combination models with upper soft rock and lower hard rock is severer than that of the combination models with upper hard rock and lower soft rock. Meanwhile, Chen et al. [30] and Liu et al. [31] found by experiments that the seismic response of slopes with thin weak interlayers is more significant than that of slopes with thick weak interlayers and that the thickness of the interlayers controls the amplification effect on the upper and lower parts of the slope, whereas the inclination angle of the interlayers controls the amplification effect on the middle and lower parts of the slope. In addition, Zhi and Yang [32] and Fan et al. [33] conducted a comparative study on the dynamic response and failure modes of bedding slopes and anti-dip slopes with weak interlayers and found that the dynamic response of the former is severer than that of the latter, with the bedding slope mainly sliding along the weak interlayers, while the weak interlayers in the anti-dip slope mainly produce compressive deformation. However, there are still many issues in the current research. For example, Cao et al. [34] found that the strong weathering zone inside the slope can significantly weaken the peak acceleration, while Liu et al. [29] found that the acceleration amplification effect in soft rock is significantly enhanced compared to hard rock. In terms of the number of soft rock layers or weak interlayers, Fan et al. [33] and Yang et al. [35] investigated the dynamic response and failure modes of bedding rock slopes containing multiple weak interlayers, and the results showed that the weak interlayers exacerbated the dynamic response of the slope, ultimately leading to sliding failure along the weak interlayers. Therefore, the existing research results show that weak interlayers have two opposite effects on seismic waves: reduction and enhancement, and their mechanisms of action are not yet clear. Meanwhile, although researchers have studied the dynamic response of slopes with different numbers of weak interlayers, they have not performed a good comparative analysis.

With respect to the consideration of earthquakes, current research has focused on the effects of factors such as seismic frequency, intensity, and direction of excitation on the seismic response of slopes. In terms of seismic frequency, Bouckovalas and Papadimitriou [36] explored parametrically the effects of slope geometry and predominant excitation frequency and duration, as well as dynamic soil properties on seismic ground motion. Lin and Wang [37] and Zhan et al. [38] found that the closer the main frequency of the input seismic wave is to the dominant frequency of the slope, the more intense the dynamic response is. As for different types of excitations, Liu et al. [29] found that the dynamic response of slopes under synthetic seismic waves is severer than that under unidirectional seismic waves. The experimental study by Fu et al. [39] suggests that the dynamic response of the slope under X-direction excitation of seismic waves is stronger than that under Z-direction excitation and that the X-direction and Z-direction of slopes with muddy interlayers exhibit a non-linear increasing trend with elevation. In contrast, Jiang et al. [40] found that the acceleration of slopes under X-direction and Z-direction excitations shows linear amplification and a “U-shaped” variation trend with elevation, respectively. From this, it can be noted that researchers have different conclusions regarding the type and direction of seismic excitation on the dynamic response of the slope.

It is clear that the dynamic response characteristics of weak interlayers under seismic action are extremely complex and influenced by many factors, and the dynamic response patterns of slopes vary in numerous experimental results. Therefore, this study uses a bedding slope-containing multiple soft rock layers in Yunnan, China as a prototype and uses the three-dimensional dynamic finite-difference method (TDD-FDM) to analyze and evaluate the dynamic response of the bedding rock slope systematically and comprehensively. The dynamic response mechanism of bedding rock slopes containing different thicknesses, locations, and quantities of soft rock layers is analyzed. The acceleration response pattern of slopes under different excitation directions of seismic waves is further explored, and the differences in the dynamic response of slopes under different excitation directions are comparatively analyzed. The research results can provide theoretical references for the study of seismic response patterns and seismic design of layered rock slopes with soft and hard rock interlayers in strong earthquake areas.

## 2. Methodology

### 2.1. Slope Models

**Figure 2:**
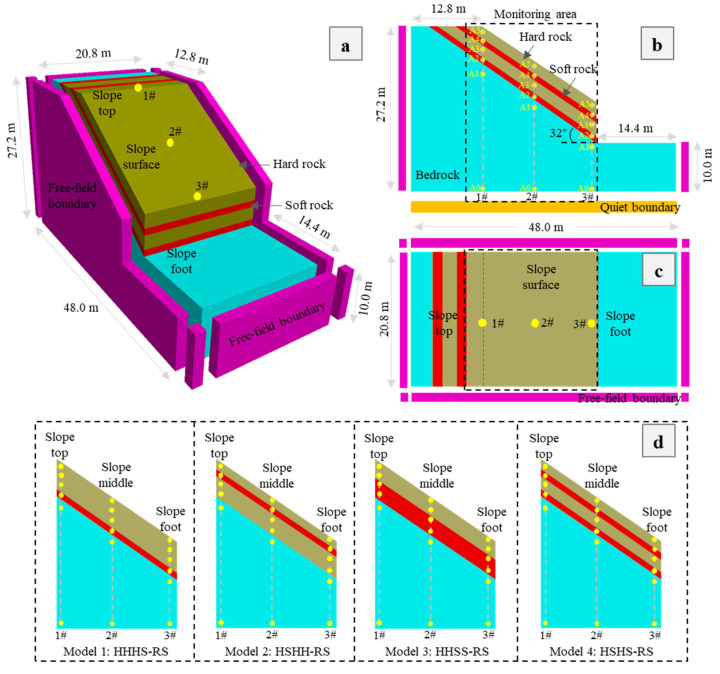
Bedding slopes with alternatively distributed soft and hard rock layers: (**a**) Numerical model, (**b**) front view, (**c**) top view, and (**d**) four slope structures.

In this study, a soft–hard interlayered bedding rock slope along a highway in Yunnan Province, China (27.0° N, 103.5° E), is used as a prototype to establish numerical models for the bedding rock slope with the pile–anchor composite structures using the TDD-FDM. The rock slope (RS) has a length of 48 m, width of 20.8 m, and height of 27.2 m, as shown in Figure 2a. The model consists of hard rock layers (H), soft rock layers (S), and bedrock layers (B), with a slope angle and layer angle of 32°. Among them, there are four rock layers covering the bedrock layer, with thicknesses of 1.76 m, 0.88 m, 1.76 m, and 0.88 m from top to bottom. According to the distribution of soft and hard rocks, four different slope structures were set up, and the corresponding numerical models are established. Model 1 (HHHS-RS) consists of a single soft rock layer of about 0.88 m thickness and a hard rock layer of about 4.40 m thickness; Model 2 (HSHH-RS) consists of a single soft rock layer and two hard rock layers, with a soft rock layer of about 0.88 m thickness and the top and bottom hard rock layers of about 1.76 m and 2.64 m thicknesses, respectively; Model 3 (HHSS-RS) consists of a single soft rock layer of about 2.64 m thickness and a hard rock layer of about 2.64 m thickness; Model 4 (HSHS-RS) consists of two layers of soft rock and hard rock, with a thickness of approximately 0.88 m for the soft rock and 1.76 m for the hard rock. The hard rock, soft rock, and bedrock of the slope are all modeled using the Mohr–Coulomb constitutive model, and the physical and mechanical parameters from the indoor tests are shown in Table 1. After the excavation of the slope, the pile–cable–beam structure is used to the slope. The pile, cable, and beam are simulated using the Pile, Cable, and Beam structural elements embedded in FLAC^3D^ [41]. Before conducting numerical simulations, the predominant frequencies of four slope models are calculated as 3.75, 3.50, 2.75, and 3.00 Hz.

### 2.2. Monitoring Points

To investigate the dynamic response patterns of stratified rock slopes under seismic conditions, three vertical monitoring lines are established at the top, middle, and foot of the slope (Figure 2b). Each monitoring line with six monitoring points is located in the four rock layers (A2–A5), the bedrock (A1), and the base of the model (A0). Using A0 as the reference point, the amplification factor of peak acceleration at different positions can be determined by Equation (1):

(1) $$F=\frac{a_{An}}{a_{A0}}$$

where *F* is the amplification factor of peak acceleration; $a_{A0}$ is the peak acceleration of the seismic wave at the model bottom A0; and $a_{An}$ is the peak acceleration of the seismic wave at bedrock, soft rock, and hard rock A1–A5.

### 2.3. Seismic Waves

**Figure 3:**
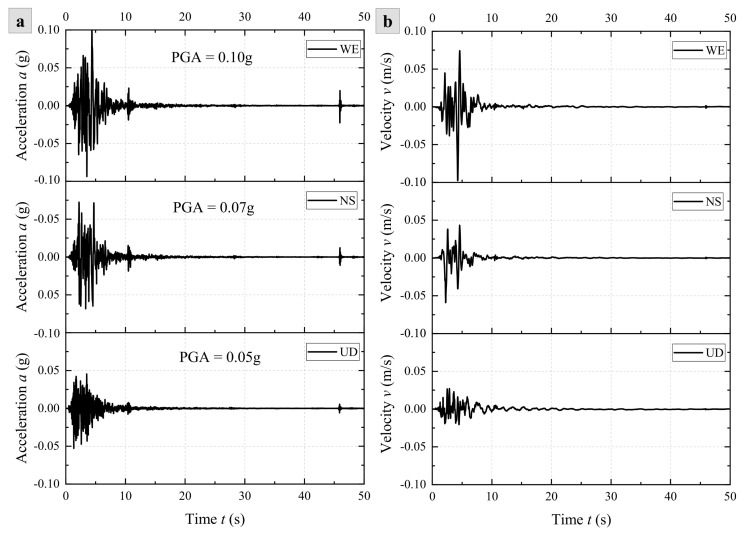
Ludian seismic wave (WE, NS, UD): (**a**) *a*–*t* curve and (**b**) *v*–*t* curve.

The seismic waves selected for this study, including WE, NS, and UD directions, were obtained at the Longtoushan monitoring station on 3 August 2014, in Ludian County, Yunnan, China (27.1° N, 103.3° E) (Figure 3). The earthquake took place with a magnitude of 6.5 and a depth of 12 km, which triggered a lot of slope instability, causing significant economic losses and casualties. The horizontal distance between the epicenter and the rock slope in this study is about 15 km. Thus, this earthquake was selected to study the dynamic response of the rock slope in this study. To assess the effect of seismic waves from different excitation directions on the rock slope dynamic responses, three seismic components are introduced as inputs at the base of the model in the horizontal X- and Y- and vertical Z-directions. Excitation directions include X, Y, Z, XY, XZ, YZ, and XYZ. Additionally, in order to investigate the effect of seismic intensity on the dynamic response characteristics of the slopes, four different peak ground accelerations (PGAs) are used in this study: 0.05 g, 0.10 g, 0.15 g, and 0.20 g.

During the dynamic calculations, owing to the quiet boundaries at the bottom of the model, seismic waves are input in the form of *σ*–*t* curves (time: 0–50 s). The process of inputting seismic wavefronts involves several steps: firstly, the acceleration–time history curve is filtered and baseline corrected; subsequently, the acceleration–time history of the Ludian wave is integrated over time to derive the velocity–time history (Figure 3b); finally, the *σ*–*t* curve is calculated using Equations (2)–(5) and then applied to the base of the model. Notably, P-waves are calculated using Equations (2) and (3), while S-waves are calculated using Equations (4) and (5).

(2) $$\sigma_{n}=-2\times \left(\rho \times C_{p}\right)\times v_{n}$$

(3) $$C_{p}=\sqrt{\frac{K+4G/3}{\rho }}$$

where $\sigma_{n}$ represents the normal force applied to the bottom boundary of the model; ρ denotes the density of the bedrock; $v_{n}$ represents the normal velocity applied to the bottom boundary of the model; $C_{p}$ represents the P-wave velocity in the bedrock; K is the volumetric deformation modulus of the bedrock; and G is the shear modulus of the bedrock.

(4) $$\sigma_{s}=-2\times \left(\rho \times C_{s}\right)\times v_{s}$$

(5) $$C_{s}=\sqrt{\frac{G}{\rho }}$$

where $\sigma_{s}$ is the tangential force applied to the bottom boundary of the model; $v_{s}$ is the tangential velocity applied to the bottom boundary of the model; and $C_{s}$ is the S-wave velocity in the bedrock.

### 2.4. Simulation Conditions

Three variables were included in this study: four slope structures, seven excitation conditions, and four kinds of seismic intensity. The specific numerical simulation conditions are listed in Table 2.

## 3. Results

### 3.1. Effect of Slope Structures

Table 3 shows the internal dynamic response of bedding rock slopes with different locations, thicknesses, and quantities of soft rock layers. The acceleration amplification factors (AAFs) of the slope show an overall increasing trend, and the maximum AAFs at the soft rock layer are significantly greater than those at the hard rock layer and bedrock layer. For example, the maximum AAFs for soft rock layers of four models are 2.15, 1.95, 2.06, and 2.22, respectively, while the maximum AAFs for hard rock layers of four models are 1.83, 2.21, 2.04, and 1.88, respectively, and the maximum AAFs for bedrock layers of four models are 1.14, 1.18, 1.18, and 1.17, respectively. Actually, the maximum AAF is not universal in the analysis of seismic response. To better compare the general patterns of acceleration amplification effects for different slope models, the average value of AAFs can be calculated as follows.

(6) $${\bar{F}}_{mp}=\frac{\sum F_{mp}}{n_{ml}\times n_{pga}}$$

(7) $$\bar{F}=\frac{{\bar{F}}_{mp}}{n_{mp}}$$

where $F_{mp}$ is the AAFs of a certain monitoring point; ${\bar{F}}_{mp}$ is the average value of all AAFs at each monitoring point; $n_{ml}$ is number of monitoring lines (1#, 2#, and 3#); $n_{pga}$ is the number of earthquake loadings; $n_{mp}$ is the number of monitoring points (A0–A5); and $\bar{F}$ is the average value of all AAFs at all monitoring points.

**Figure 4:**
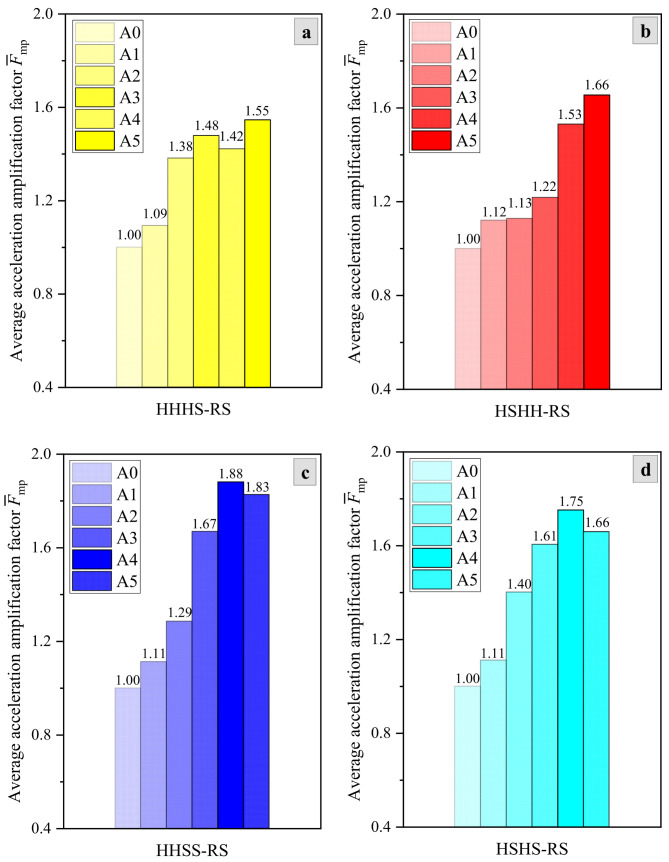
Dynamic response of bedding rock slopes with soft rocks of different characteristics: (**a**) HHHS-RS, (**b**) HSHH-RS, (**c**) HHSS-RS, and (**d**) HSHS-RS.

Figure 4 shows that the maximum values of ${\bar{F}}_{mp}$ of the four slope models (HHHS-RS, HSHH-RS, HHSS-RS, and HSHS-RS) are 1.55, 1.66, 1.88, and 1.75, respectively. Comparing the AAFs of the models, it can be found that acceleration amplification effect of the slope is severer when the soft rock layer is located in the upper part than that in the lower part, and the thicker the soft rock layer is, the stronger the amplification effect is. Furthermore, the acceleration amplification effect of the slopes containing two layers of soft rock is severer than that of the slopes containing one layer of soft rock. Thus, with regard to the slope structures, the order of factors for the influence of the soft rock layer on seismic response in bedding rock slopes with alternatively distributed soft and hard rock layers is thickness > quantity > location.

### 3.2. Effect of Excitation Directions

**Figure 5:**
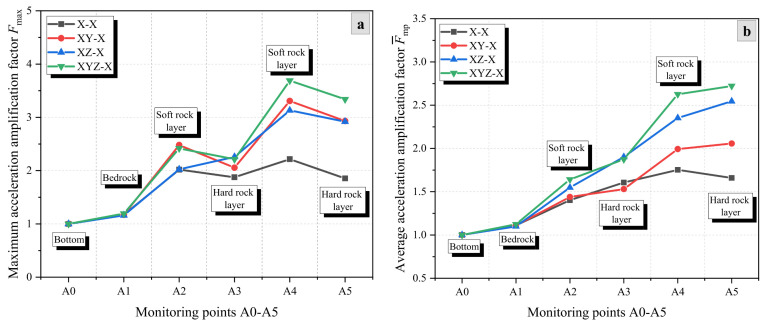
AAFs in the X-direction of the slope under different excitation conditions: (**a**) $F_{max}$ and (**b**) ${\bar{F}}_{mp}$.

**Figure 6:**
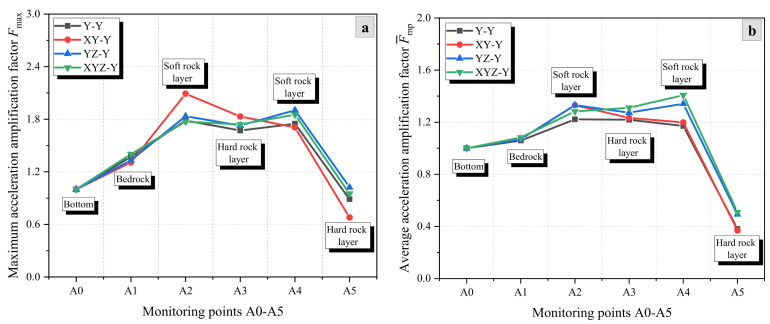
AAFs in the Y-direction of the slope under different excitation conditions: (**a**) $F_{max}$ and (**b**) ${\bar{F}}_{mp}$.

**Figure 7:**
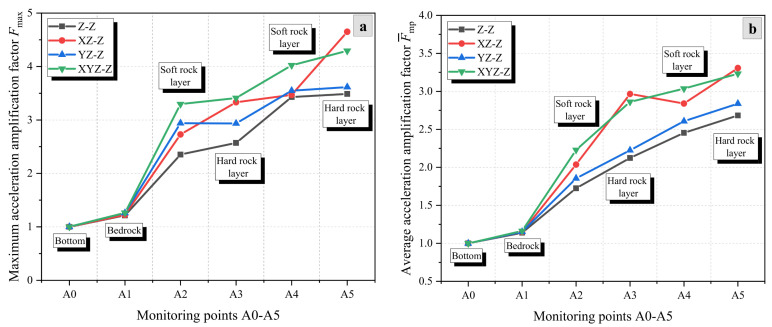
AAFs in the Z-direction of the slope under different excitation conditions: (**a**) $F_{max}$ and (**b**) ${\bar{F}}_{mp}$.

Figure 5, Figure 6 and Figure 7 show the variation of the AAFs in the X-, Y-, and Z-directions of the rock slope (HSHS-RS) with increasing elevation under different excitation conditions of seismic waves. It can be observed that the AAFs in the X- and Z-directions show an overall increasing trend with elevation, while the AAFs in the Y-direction shows a trend of first increasing and then decreasing. Furthermore, the acceleration amplification effect in the Z-direction is greater than that in the X- and Y-directions. For example, under unidirectional excitation, the maximum AAFs, $F_{max}$, of the three directions are 2.22, 1.79, and 3.49, respectively (Figure 5a, Figure 6a and Figure 7a).

Under unidirectional (X), bidirectional (XY and XZ), and tri-directional (XYZ) excitation directions of seismic waves, the maximum and average AAFs of the X-direction show a general increase with increasing elevation (Figure 5). Specifically, under bidirectional and tri-directional excitations, the AAFs of the X-direction show a stepped increasing trend, indicating a strong amplification effect in soft rock layers and a weak amplification effect in hard rock layers. Under the four excitation conditions, the average AAFs, $\bar{F}$, of X-direction acceleration calculated from ${\bar{F}}_{mp}$ are 1.42, 1.52, 1.74, and 1.83, respectively (Figure 5b). The amplification effects under the four excitation conditions are ranked from high to low as XYZ, XZ, XY, and X. From this, it can be argued that compared to the unidirectional excitation condition, increasing the number of excitation directions can enhance the seismic response in the X-direction of the slope, and the enhancement effect of seismic waves in the Z-direction is much higher than that of seismic waves in the Y-direction.

Figure 6 shows the variation of the maximum and average AAFs of the Y-direction with elevation under unidirectional (Y), bidirectional (XY and YZ), and tri-directional (XYZ) excitation directions of seismic waves. It can be seen that the maximum and average AAFs exhibit a trend of first increasing and then decreasing, and the minimum value is less than 1. The maximum AAFs occur in soft rock layers, and the average AAFs $\bar{F}$ of Y-direction acceleration calculated from ${\bar{F}}_{mp}$ are 1.01, 1.03, 1.08, and 1.10, respectively (Figure 6b). The amplification effects under the four excitation conditions are ranked from high to low as XYZ, YZ, XY, and Y. This can indicate that increasing the number of excitation directions enhances the Y-direction seismic response of the slope compared to the unidirectional excitation condition, and the enhancement of the Z-direction seismic wave is much higher than that of the X-direction seismic wave.

Figure 7 shows the variation of AAFs of the Z-direction with elevation under unidirectional (Z), bidirectional (XZ and YZ), and tri-directional (XYZ) excitation directions of seismic waves. Similar to the dynamic response of X-direction acceleration, the maximum and average AAFs of the Z-direction show a continuous increasing trend. But in contrast, the maximum AAFs appear at the upper hard rock layer, which are 3.49, 4.65, 3.62, and 4.29, respectively (Figure 7a). In addition, under the four excitation conditions, the average AAFs $\bar{F}$ of Z-direction acceleration calculated from ${\bar{F}}_{mp}$ are 1.85, 2.22, 1.95, and 2.25, respectively (Figure 7b). The amplification effects under the four excitation conditions are ranked from high to low as XYZ, XZ, YZ, and Z. As a result, it can be found that the increase of an excitation direction can enhance the Z-direction seismic response of the slope, and the enhancement effect of the X-direction seismic wave is much higher than that of the Y-direction seismic wave.

Based on the above results, there are two phenomena of acceleration amplification effects on rock slopes under different seismic wave excitation directions and types: differential amplification and coupling amplification. The phenomenon of differential amplification is mainly manifested in the different patterns and degrees of acceleration amplification. The AAFs in the X-direction shows a stepped upward trend with elevation; the AAFs in the Y-direction shows a trend of first increasing and then decreasing; and the AAFs in the Z-direction shows a continuous rising trend with elevation. The acceleration amplification effect of the Z-direction is stronger than that of the X-direction and Y-direction. In addition, the phenomenon of coupling amplification is mainly manifested in the different degrees of seismic response and enhancement effects of the slope under multi-directional seismic waves. The seismic response of triaxial seismic waves (XYZ) are higher than bidirectional seismic waves (XZ, YZ, and XY) and stronger than unidirectional seismic waves (Z, X, Y). In terms of enhancing the seismic response degree, the Z-direction is stronger than the X-direction and the Y-direction. Therefore, considering the differential and coupled amplification response of natural seismic waves in the slope, the tri-directional excitation of seismic waves should be used to obtain the true dynamic response patterns of slopes. This is of great significance for the evaluation of the stability of rock slopes in strong earthquake areas and the seismic design of reinforcement projects in preventing excessive or insufficient reinforcement.

### 3.3. Effect of Seismic Intensity

In order to investigate the effect of seismic intensity, the average AAFs ${\bar{F}}_{a}$ of the layered rock slope under different seismic intensities (PGAs = 0.05–0.20 g) are further calculated as Equation (8).

(8) $${\bar{F}}_{a}=\frac{\sum F_{a}}{n_{mp}\times n_{ml}}$$

where $F_{a}$ is the AAFs of all monitoring lines of a certain model under a certain seismic loading (0.05 g, 0.10 g, 0.15 g, and 0.20 g) or the AAFs of all monitoring lines of the HSHS-RS model under a certain seismic excitation direction (X-X, XY-X, XZ-X, and XYZ-X) and ${\bar{F}}_{a}$ is the average AAFs on all monitoring lines of a certain model under a seismic loading or the average AAFs on all monitoring lines of the HSHS-RS model under a certain seismic excitation direction.

**Figure 8:**
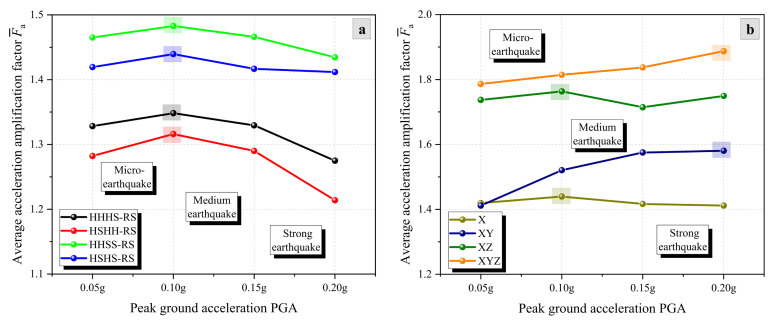
Dynamic response of bedding rock slopes under different PGAs: (**a**) Modeling factor and (**b**) excitation factor.

Figure 8 shows the dynamic response of bedding rock slopes under different PGAs. As the seismic intensity increases, the average AAFs, ${\bar{F}}_{a}$, of HHHS-RS, HSHH-RS, HHSS-RS, and HSHS-RS models show an increasing–decreasing trend (Figure 8a). This indicates that there is a significant difference in the seismic response of slopes within the PGA range of 0.05–0.20 g. Under micro-seismic conditions of 0.05–0.10 g, the overall integrity of the slope is better, and the seismic response is severer. As the seismic intensity increases, the slope is severely damaged, and the degree of seismic response gradually decreases in medium and strong earthquakes (0.10–0.20 g). In addition, as the seismic intensity increases, the ${\bar{F}}_{a}$ of the slope under X-direction and XZ-direction excitations shows an increasing–decreasing trend, while that of the slope under XY-direction and XYZ-direction excitations shows an overall increasing trend. This indicates that the incorporation of Y-direction seismic waves can change the vibration response pattern of slopes.

Based on the above results, the seismic response degree of soft–hard interlayered bedding rock slopes is not only related to the rock layer structures of the slope and excitation conditions of a seismic wave but also affected by the intensity of input seismic waves.

## 4. Discussion

The above results show that the seismic response of soft–hard interlayered bedding rock slopes in an earthquake mainly exhibits an acceleration amplification effect of soft rock, differential amplification effect, and coupling amplification effect under multi-directional seismic wave excitation. This indicates that the seismic response of soft–hard interlayered bedding rock slopes is related not only to the rock layer structure of the slope and the type of seismic wave excitation but also to the intensity of input seismic waves. However, the findings of this study are not entirely consistent with previous studies. Therefore, this section provides an in-depth comparative analysis and discussion.

**Figure 9:**
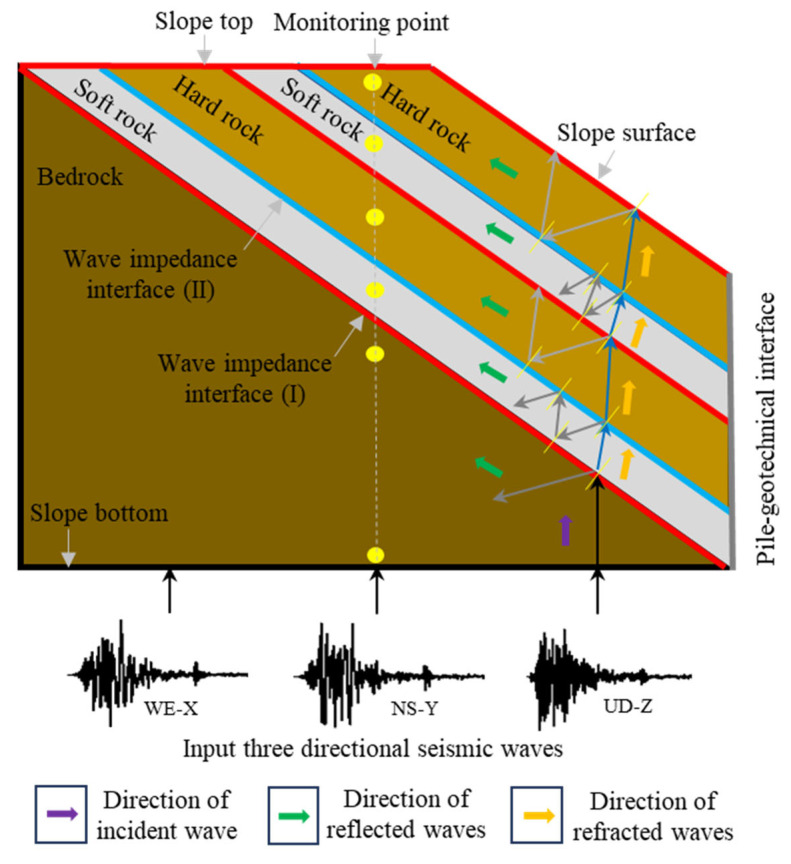
Schematic diagram of seismic wave propagation paths in different geotechnical media.

The propagation characteristics of seismic waves are closely related to the dynamic properties of the medium. When passing through different propagation media, the propagation path and intensity of the waves vary due to the existence of wave impedance interfaces. According to the theory of elastic waves [42], seismic waves vertically incident from the base are first reflected and refracted when passing through different rock interfaces and then undergo multiple reflections and refractions within the same rock layer, where various types of seismic waves are eventually superimposed to form a complex wavefield. It is generally recognized in some studies that the reflection coefficient of the reflection interface of the propagation medium is the main factor affecting the propagation of seismic waves [43,44]. However, this study infers that the propagation direction of seismic waves passing through reflection interfaces is also an important factor, namely two propagation directions of the same reflection interface (soft rock to hard rock, hard rock to soft rock). As reported by Cao et al. [34], seismic waves are believed to reflect and refract after traversing highly weathered zones. Upon transitioning from “hard rock” to “soft rock”, the dispersion of the waves diminishes the peak amplitude. Conversely, upon transitioning from “soft rock” to “hard rock”, the superposition of the waves amplifies the peak amplitude. Based on this, this study further substantiates this perspective, as illustrated in Figure 9. Seismic waves primarily traverse the wave impedance interface (I) as refracted waves, passing from bedrock into soft rock and simultaneously generating weaker reflected waves. During this phase, little change is observed in the AAFs at the monitoring point in the bedrock. Subsequently, the seismic waves entering the soft rock undergo further refraction, traverse the impedance interface to reach the hard rock, and experience multiple reflections within the soft rock. Consequently, the superposition of refracted and multiply reflected waves greatly complicates the wavefield within the soft rock layers, significantly amplifying the acceleration. The seismic waves then propagate as refracted waves into the hard rock layers, where they also undergo reflection, albeit with diminished intensity. Thus, in hard rock layers compared to soft ones, the rate of increase in the AAFs is reduced, indicating a significant attenuation of seismic wave energy. During the subsequent propagation of seismic waves, as they traverse soft and hard rocks again, their behavior mirrors that of bedrock–soft rock–hard rock, with soft rock enhancing and hard rock diminishing the seismic wave energy. This results in a stepwise increase trend in the AAFs (Figure 4 and Figure 5).

**Figure 10:**
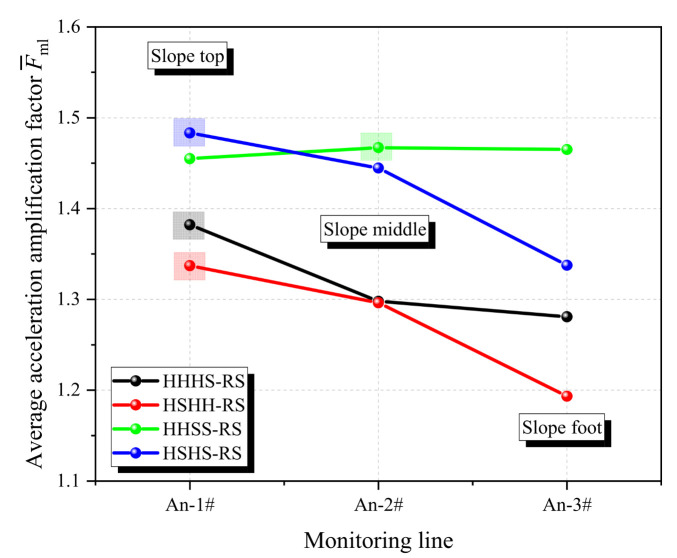
Dynamic response of bedding rock slopes for different monitoring lines.

Figure 9 also shows reflection waves in both soft and hard rock layers propagate upward along the rock layers, whereas refracted waves travel almost vertically upwards. Consequently, this results in a more pronounced superposition of various seismic wave fields in the middle and upper regions of the slope, leading to a more pronounced acceleration amplification effect compared to the slope’s lower section. To confirm this speculation, the average AAFs, ${\bar{F}}_{ml}$, at the same monitoring line inside the rock slope are further calculated as Equation (9), and its dynamic response pattern is presented in Figure 10. Figure 10 shows the average AAFs at different locations within the four slope models. Among them, the average AAFs of HHHS-RS, HSHH-RS, and HSHS-RS models indicate that the seismic response in the upper part of the slope is the strongest, while the average AAFs of the HHSS-RS model indicate that the seismic response in the middle part of the slope is the strongest. There are significant differences in the seismic response patterns at the top, middle, and foot of the slope, which provides preliminary evidence of the directional propagation of seismic waves in different rock media.

(9) $${\bar{F}}_{ml}=\frac{\sum F_{ml}}{n_{mp}\times n_{pga}}$$

where $F_{ml}$ is the AAFs on a certain monitoring line of a certain model under all seismic loadings (0.05 g, 0.10 g, 0.15 g, and 0.20 g) and ${\bar{F}}_{ml}$ is the average AAFs on a certain monitoring line of a certain model under all seismic loadings.

**Figure 11:**
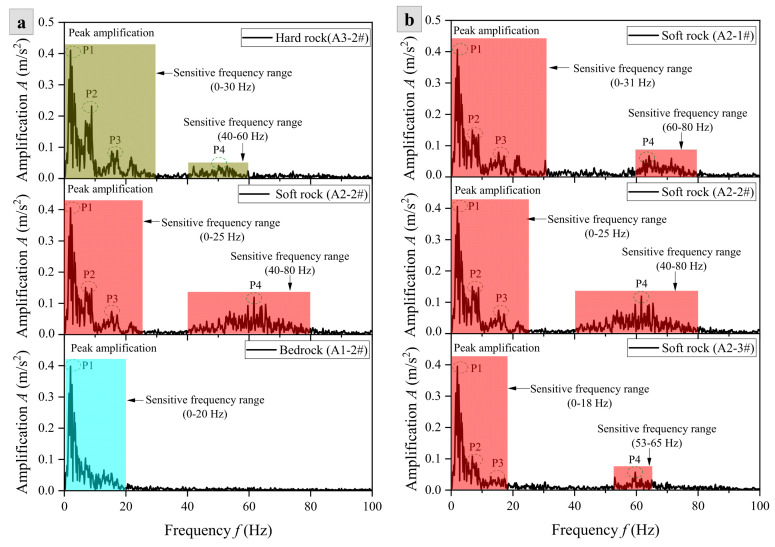
Amplification–frequency curves of rock slopes under X-direction seismic excitation of PGA = 0.10 g: (**a**) Different rock layers and (**b**) different monitoring lines.

Meanwhile, the analysis of the spectra of bedrock, soft rock, and hard rock provides good evidence for the above viewpoint (Figure 11). As shown in Figure 11a, during the propagation of seismic waves from bedrock (A1-2#) to soft rock (A2-2#), the amplification of fixed frequency bands significantly increases, with multiple peak amplitudes appearing (P1–P4). The length of the first sensitive frequency band increases (0–25 Hz), and the number of sensitive frequency bands increases (40–80 Hz). When seismic waves propagate from soft rock (A2-2#) to hard rock (A3-2#), the length of the first sensitive frequency band increases (0–30 Hz), while the length of the second sensitive frequency band decreases to 40–60 Hz, and the peak value decreases from 0.120 m/s^2^ to 0.042 m/s^2^. This is because the structural characteristics of the soft–hard interlayered rock slope itself are more sensitive to a specific frequency band in seismic waves, and the sensitivity of soft rock to this frequency band is higher than that of hard rock, making the amplification effect more significant. Especially by increasing the thickness and number of soft rock layers, it is more likely to cause severe vibration of the soft rock layer, leading to serious failure of the slope. Meanwhile, Figure 11b shows that from the foot to the top of the slope, the length and peak values of the sensitive frequency bands in the middle (A2-2#) and top (A2-1#) of the slope are significantly greater than those in the foot (A2-3#). Among them, the first sensitive frequency band lengths of A2-1#, A2-2#, and A2-3# are 0–18 Hz, 0–25 Hz, and 0–31 Hz, respectively, with peak values of 0.395, 0.406, and 0.407 m/s^2^, respectively; the second sensitive frequency bands are 53–65 Hz, 40–80 Hz, and 60–80 Hz, with peak values of 0.057, 0.120, and 0.068 m/s^2^, respectively. This proves that the superposition of various seismic wave fields in the upper part of the slope is the most significant, and the seismic response is the severest.

In addition, by comparing the dynamic response of bedding slopes with different slope structures, this study suggests that when soft rock layers are located at the upper part, the degree of dynamic response of the slope is more intense than when they are located at the lower part, which is consistent with previous studies [9,29]. In terms of the thickness of soft rock layers, this study suggests that the dynamic response degree of slopes with thick, soft rock layers is higher than that of slopes with thin, soft rock layers. However, Liu et al. [31] have obtained an opposite result, suggesting that horizontal thick, soft intercalations have a weakening effect on seismic waves. It is speculated that this may be related to the predominant frequencies of a rock slope. In this study, the dominant frequency of the input WE component of the Ludian earthquake wave is 1.62 Hz. It can be observed that the order of difference between the predominant frequencies for the four models and the dominant frequency of seismic waves from small to large is HHSS-RS (2.75 Hz), HSHS-RS (3.00 Hz), HSHH-RS (3.50 Hz), and HHHS-RS (3.75 Hz). This is consistent with the ranking of the dynamic response degree of the four models in an earthquake. It can be found that the closer the predominant frequency of a slope is to the dominant frequency of a seismic wave, the more intense its dynamic response will be. This has also been mentioned and proven in the study by Zhan et al. [38].

Finally, comparing the results of the dynamic response of a slope under different excitation directions, it is found that there are differential amplification and coupling amplification of acceleration. Among them, the seismic response degree under multidirectional seismic waves is more intense than that under unidirectional seismic waves, which is also confirmed in the study by Liu et al. [29]. However, some research results are quite different from previous studies. For example, in this study, the AAFs in the Z-direction show an increasing trend with elevation, while in the study by Jiang et al. [40], the Z-direction show a “U” trend with elevation. The maximum value of the three-dimensional AAFs in this study are in the order of Z > X > Y, while in the study by Fu et al. [39], the AAFs in the X-direction are larger than those in the Z-direction. The input seismic wave in this study is the Ludian wave, while previous studies have mostly input the Wenchuan wave. Therefore, it is speculated that the different research results may be due to the different types of seismic waves.

To sum up, the seismic response degree of a soft–hard interbedded bedding rock slope in an earthquake is affected by the slope structure and seismic wave. Firstly, the rock structure inside the slope mainly includes the strength of rock mass, location, thickness and number of soft rock layers. Therefore, in the design of seismic reinforcement structures for soft–hard interbedded bedding rock slopes, the slope structure and lithological characteristics of the on-site slope should be fully obtained through methods, such as drilling and indoor testing, to comprehensively evaluate and judge the stability of the slope under seismic loads based on the dynamic response of the slope. Meanwhile, the seismic wave includes the type, strength, and excitation direction of the input seismic wave. Especially, the phenomena of acceleration differential and coupling amplification caused by the triaxiality of natural seismic waves have a significant impact on the seismic reinforcement design of on-site slopes. Therefore, as conducting model experiments and numerical simulation studies, three-dimensional excitation should also be carried out to more accurately analyze the acceleration amplification effect of slopes.

## 5. Conclusions

To investigate the dynamic response of rock slopes containing soft rock under seismic loads, several simplified bedding slope models were established using the TDD-FDM, based on the prototype of a bedding rock slope in Yunnan, China. The dynamic response mechanism of bedding rock slopes with different thickness, location, and quantity of soft rock layers was studied, and the acceleration response under different excitation directions of seismic waves was further explored; and the difference of the dynamic response of rock slopes under different excitation directions was compared and analyzed. The main findings are as follows:

(1) The propagation of seismic waves in different rock layer structures has directionality, which causes the strongest seismic response of HHHS-RS, HSHH-RS, HHSS-RS, and HSHS-RS is all located in the upper or middle parts of the slope. The maximum values of average AAFs of the four slope models in an earthquake are HHSS-RS > HSHS-RS > HSHH-RS > HHHS-RS, which indicates that the dynamic response of rock slopes is stronger as the soft rock layers become lower in location, more numerous, and thicker in layer.

(2) Two phenomena of the dynamic response of slopes under different seismic wave excitation: differential amplification and coupling amplification. The former is mainly manifested by the fact that the AAFs in X-direction and Z-direction increase with elevation, while the AAFs in Y-direction increase first and then decrease, and the order of the acceleration amplification degree is Z-direction, X-direction, and Y-direction. The latter is mainly manifested by the fact that the order of the dynamic response degree of seismic wave excitation types is the tri-directional, bidirectional, and unidirectional excitations, and the order of enhanced dynamic response is the Z-, X-, Y-directions.

(3) The average AAFs of slopes under different PGAs show two trends: increasing–decreasing and overall increasing. The integrity of the rock mass inside the slope is good under micro-seismicity, but as the seismic intensity increases, the structure of the rock mass is damaged. This leads to average AAFs of the rock slope showing an increasing–decreasing trend. However, comparing different excitation directions, the Y-direction can reduce the seismic response in the X- and Z-directions, which changes the trend from first increasing and then decreasing to continuously increasing.

## Figures and Tables

**Table 1:** Physical and mechanical parameters of geotechnical bodies of the slope.

Index	Lithology		
	Hard Rock	Soft Rock	Bedrock
Density *ρ* (kg·m^−3^)	2470	2070	2470
Elastic modulus *E* (GPa)	5.92	0.065	5.92
Poisson’s ratio *μ*	0.22	0.33	0.22
Cohesion *c* (MPa)	7.52	0.21	7.52
Internal friction angle *φ* (°)	31	22	31

**Table 2:** The simulation conditions in this study.

Slope Structures					Excitation Direction and Intensity		
No.		*Q*_s_	*T*_s_	*L*_s_	X	Y	Z
Model 1	HHHS-RS	1	0.88 m	Lower	0.05–0.20 g	/	/
Model 2	HSHH-RS	1	0.88 m	Upper	0.05–0.20 g	/	/
Model 3	HHSS-RS	1	2.64 m	Lower	0.05–0.20 g	/	/
Model 4	HSHS-RS	2	0.88 m	/	0.05–0.20 g	/	/
Model 4	HSHS-RS	2	0.88 m	/	/	0.05–0.20 g	/
		2	0.88 m	/	/	/	0.05–0.20 g
		2	0.88 m	/	0.05–0.20 g	0.05–0.20 g	/
		2	0.88 m	/	0.05–0.20 g	/	0.05–0.20 g
		2	0.88 m	/	/	0.05–0.20 g	0.05–0.20 g
		2	0.88 m	/	0.05–0.20 g	0.05–0.20 g	0.05–0.20 g

Notation: H—Hard rock layer; S—Soft rock layer; RS—Rock slope; *Q*_s_—Quantity of soft rock layers; *T*_s_—Thickness of soft rock layers; *L*_s_—Location of soft rock layers; X—X-direction acceleration; Y—Y-direction acceleration; and Z—Z-direction acceleration.

**Table 3:** AAFs of the four slope models in an earthquake in this study.

No.	Monitoring Points	Slope Top (1#)				Slope Middle (2#)				Slope Foot (3#)			
		0.05 g	0.10 g	0.15 g	0.20 g	0.05 g	0.10 g	0.15 g	0.20 g	0.05 g	0.10 g	0.15 g	0.20 g
HHHS-RS	A0	1.00	1.00	1.00	1.00	1.00	1.00	1.00	1.00	1.00	1.00	1.00	1.00
	A1	1.14	1.12	1.01	1.14	1.12	1.02	1.14	1.11	1.03	1.13	1.12	1.03
	A2	1.51	2.15	1.40	1.22	1.68	1.16	1.27	1.40	1.10	1.34	1.26	1.09
	A3	1.39	1.77	1.70	1.30	1.49	1.62	1.34	1.42	1.51	1.29	1.57	1.33
	A4	1.38	1.60	1.52	1.41	1.42	1.30	1.37	1.48	1.40	1.34	1.52	1.34
	A5	1.52	1.55	1.83	1.51	1.42	1.40	1.49	1.50	1.80	1.48	1.39	1.67
HSHH-RS	A0	1.00	1.00	1.00	1.00	1.00	1.00	1.00	1.00	1.00	1.00	1.00	1.00
	A1	1.13	1.18	1.07	1.15	1.16	1.05	1.15	1.14	1.05	1.15	1.17	1.04
	A2	1.13	1.14	1.24	1.14	1.13	1.08	1.15	1.13	1.07	1.14	1.14	1.06
	A3	1.16	1.30	1.55	1.16	1.31	1.26	1.16	1.24	1.07	1.16	1.17	1.07
	A4	1.60	1.95	1.82	1.37	1.75	1.77	1.33	1.34	1.35	1.33	1.48	1.29
	A5	1.98	1.62	1.81	1.59	1.66	2.21	1.45	1.64	1.53	1.35	1.53	1.49
HHSS-RS	A0	1.00	1.00	1.00	1.00	1.00	1.00	1.00	1.00	1.00	1.00	1.00	1.00
	A1	1.15	1.15	1.03	1.18	1.14	1.03	1.17	1.14	1.04	1.17	1.11	1.03
	A2	1.31	1.27	1.08	1.44	1.30	1.09	1.33	1.28	1.19	1.58	1.38	1.16
	A3	1.47	1.58	1.96	1.45	1.68	1.69	1.56	1.55	2.06	1.70	1.74	1.59
	A4	1.94	1.93	1.75	1.90	1.91	1.97	1.73	1.98	1.73	2.04	1.93	1.76
	A5	1.90	1.84	1.81	1.76	1.95	1.77	1.96	1.98	1.60	1.88	1.85	1.62
HSHS-RS	A0	1.00	1.00	1.00	1.00	1.00	1.00	1.00	1.00	1.00	1.00	1.00	1.00
	A1	1.17	1.13	1.06	1.17	1.13	1.04	1.17	1.12	1.03	1.16	1.11	1.04
	A2	1.58	2.02	1.13	1.59	1.70	1.11	1.56	1.41	1.08	1.29	1.26	1.09
	A3	1.73	1.56	1.54	1.77	1.55	1.56	1.77	1.67	1.48	1.88	1.31	1.44
	A4	1.76	1.65	1.93	1.87	1.76	1.72	2.22	1.61	1.55	1.78	1.58	1.60
	A5	1.79	1.85	1.63	1.67	1.72	1.58	1.56	1.71	1.52	1.58	1.67	1.65

## Data Availability

The original contributions presented in the study are included in the article, further inquiries can be directed to the corresponding author.
